# Hygienic Laws

**Published:** 1876-10

**Authors:** G. W. Klump

**Affiliations:** WILLIAMSPORT.


### AMERICAN JOURNAL OF DENTAL SCIENCE.

Vol. X. THIRD SERIES—OCTOBER, 1876. No. 6.

### ARTICLE I.

### *Hygienic Laws.*

BY G. W. KLUMP, D. D. S., WILLIAMSPORT.

GENTLEMEN : It is not my custom to make apologies when I have a duty assigned me to perform, and yet I must be permitted to ask the indulgence of this audience for the imperfect manner in which I bring my views before this society.

My only excuse is languidness and ill health, caused by a violation of those very laws of which I propose to treat. Nevertheless, I hope I may be able to show you the great importance and necessity of properly observing and obeying them.

Hitherto it has seemed to me that nearly all our efforts have been made to advance the art and science of dentistry, for the purpose of seeing how much we could do to benefit those who are placed under our professional care, regardless of our own health, happiness and comfort; overlooking the laws of sanitary science, and infringing them in our earnest zeal to benefit our patients, not knowing, or at least not seeming to realize, that whatsoever we do that causes deterioration of our own health, detracts correspondingly not only from our own comfort and happiness, but also from rendering to our patients the best professional services we would otherwise be capable of. So much have I been impressed with this erroneous direction of the efforts among the great mass of our profession, in their endeavors to advance it to the dignity and importance it commands, that I have been persuaded to write upon it, not for the purpose of throwing any great light upon it, but more especially to direct your attention to this subject, and have it thoroughly discussed by those who are more familiar with it, and better able to enlighten you in regard to it.

We learn by observation that every created being is subjected to certain fixed laws by the divine Maker, immutable in their results, which, when properly observed, promote his happiness and well-being, but if violated, cause pain, disease, and finally death. By the law of gravitation, for instance, the body falls to the ground, unless properly supported, and is liable to injury. By chemical laws excessive heat dissipates the fluids of the body ; and in either case injury and death ensue. Transgressing the laws of light, electricity and ventilation, though perhaps not so manifest to our senses, is equally injurious to life, health and happiness.

We see, then, as physical beings we are subject to physical laws, which are fixed and invariable ; and, although many of them are imperfectly understood, and others, in all probability, as yet entirely unknown ; yet the consequences are the same, whether they be ignorantly violated or knowingly transgressed.

The law of sunshine, for example, gives light, alike to the just and unjust. The law of water, while it floats to the destruction of some peaceable town, carries as well the iron-clad monster of war, as the pleasure yacht filled with the gay and joyous; not because the mission is either to destroy or take pleasure, but because the part of the ship being immersed displaces a weight of water equal to its whole weight, leaving the remaining portion above the fluid. A ship, therefore, will float as long as these conditions are observed.

So, also, the man who swallows poison will die, because an organic law has been infringed; whether he has taken the poison intentionally, by mistake, or through ignorance. We see, then, that these natural laws are universal and invariable, and when properly understood are in entire harmony with the constitution of man. But how are we to comprehend these laws? I answer by having a perfect knowledge of the laws by which all things which God has made are governed. But is this attainable? Are these laws within our grasp? Is this world, which we are taught was made for man, *really* his? Or is it his only in the same sense as it is of every living thing that moves upon it—a temporary home, a mere spot to live on, but intended for some higher race, and governed by laws that are too sublime for our feeble intellects. Laws that *appear* to minister to our wants, that *appear* to be wielded by us for our purposes, but which are set above our reach, forever mocking us with their unapproachable grandeur. If so, then are we not, of all things, most miserable? The plodding industry which knows no rest in pursuit of its object, and the soaring genius that sweeps to the very sun in its daring flights, are alike the veriest folly that can be conceived of. But is it not so? "We are monarchs of all we survey. Our rights there are none to dispute." Our right to know and to rule comes with our life from God himself. The human intellect is gifted with powers and faculties large enough and strong enough to grasp and comprehend these physical laws, and to make use of them for its own purposes. This is the distinguishing insignia of our race. With these powers, it follows that the future of our race, *even here* is without limit, if properly exercised. On everything that

God has made he has impressed certain laws, on which their very existence depends. Under favorable circumstances, or, in other words, when the laws peculiar to each particular thing have free action, it is found to flourish: when the opposite is the case, it fails.

To know these laws is to be practically learned; not to know them is ignorance: and in our dealings with the things which God has placed around us, we are strong and successful, or weak and valueless, just as we know or do not know the peculiar laws which he has impressed upon them. With the untamed and uneducated savage, as with us, the laws of nature are the same. Gravitation, inert motion, are alike everywhere, in every kingdom of nature in air, and earth, and sky still the same; simple, unchanging and unchangeable. But with *them* the waterfall makes its power to no purpose; the planets move in their appointed spheres, apparently aimless and useless; the sun shines, the rains fall, and the winds blow as they list; the streams wend their silent, unobstructed way from hill to vale, and from the valley to the sea; the lightning flashes tameless and untamable from cloud to cloud, marking its career with terror and desolation, and animated nature owes no allegiance to a being no wiser than itself.

Thus was the world for ages, until the laws which govern it were understood by man. Then was it brought into subjection to him. The water no longer tumbled down the cataract in idleness; the winds no longer roamed over land and sea; but the one was made to turn the ponderous wheel or was compelled to bear upon its bosom the wealth of commerce. The planets were made the landmarks of the vast career, and the lightning tamed and taught to weld the gold with lightning strokes, or flashes the lightest whisper round the globe.

Thus our powers ever are and ever will be in proportion to our knowledge. Our power over matter is obtained by a knowledge of the laws which govern it. If the laws which regulate *Health* and *disease* are any *less* under our control, it is not because they are not less fixed and certain in their results, but only because they are not so well known and understood. The time is past for believing that our sickness and afflictions are sent, not brought, upon ourselves. When a deadly epidemic sweeps over the land, depopulating its inhabitants, it is no more regarded as a frown of the Deity, or as a judgement sent from God upon us, by persons conversant with the laws of sanitary science.

These laws, if properly observed, always lead to health and happiness; but when they are disregarded and disobeyed, the evil effects will as certainly follow. When we expose ourselves to excessive heat or cold, we suffer pain. So, also, when we neglect proper exercise, or eat our food hastily, imperfectly masticating it, with insufficient rest before or after meals, we soon suffer from indigestion and its consequent train of evils. Thus we are admonished under penalty of pain, disease and death, not to disobey the laws of sanitary science.

If these gentle admonitions were properly heeded, and these laws, with those of heat, light and ventilation, and all the hygienic and sanitary measures which we, as dentists, are so particularly inclined to disobey, were more strictly observed, we should have more robust, healthy men in our profession, and fewer broken down, disabled dentists.

The first important sanitary measure to observe, is to select a place of business in a healthy locality, not subject to malaria, nor poisonous gases, from defective sewers or other sources; and the next, of no less importance, is to have a well-lighted affice, with means of perfect ventilation which should depend not merely upon the lowering or raising of one or two windows, but every room should have ventilating flues opening near the ceiling into the room; this, together with the fresh air ducts, would ordinarily insure a perfect displacement of the vitiated atmosphere, so essential to health and life.

Another means, more certain in its results, is to have an open fire-place with fire in the room. It is even more important to have pure air for the lungs than it is to have pure water to drink or pure food to eat. The horrors of the "Black Hole of Calcutta" are familiar to every on e yet the same thing is, in a variety of ways, to a great extent, constantly repeated without protest.

Pure air is a most excellent tonic—the very best blood-purifier, as well as the best preventive of disease. What an army of pale, feeble, emaciated sufferers might be restored to health and comfort by taking into their starving, poisoned lungs pure air—nature's restorer—and discarding the drugs and stomach bitters upon which quacks thrive, while their patients die. When we consider that fully forty per cent. of all deaths are caused by foul air alone, we at once see the great necessity of improving our architecture, so as to prevent this suicidal record. Experiments prove that carbonic acid gas is not the only or even worst poison which proceeds from the human body, and which it is necessary to remove by thorough ventilation.

There are also many gases deleterious to health generated in the dentist's laboratory, while performing the various mechanical and chemical operations; and these mephitic gases permeate the operating and reception rooms also with their pernicious influence, when they are connected with the laboratory, as is frequently the case. The better way to avoid the evil consequences arising from these poisonous gases, is to have pipes to carry them directly away, besides having every room constantly and thoroughly ventilated. Dentists are perhaps, compelled more or less to breathe impure exalations from their patients, on account of the necessary close proximity during many operations; and they should use all the available means to counteract and neutralize them, well knowing that nearly all the diseases of the lungs are caused by breathing impure air, and that it is quite as important to guard the lungs against appropriating impure food as it is to guard the stomach against taking poisonous adulterations.

Light, that boon from heaven, that those only hate whose deeds are evil, also plays a most important part in the promotion of health. The total exclusion of light causes impoverished blood, impaired vitality and a general physical, mental, and even moral debility. The rays of the sun have also the power of purifying the air, which can be readily seen by noticing the difference in the air of a room kept constantly dark and one well lighted, however close it may otherwise be. Light is not only essential in preventing disease, but also acts as a healthy stimulus, in many instances, in treating disease. Sir David Brewster refers to this subject in the following language: "If the light of day contributes to the development of human form, and lends its aid to art and nature in the cure of disease, it becomes a personal and national duty to construct our dwelling houses, schools, workshops, factories, churches, villages, towns and cities upon such principles and in such styles of architecture as will allow the life-giving element to have the fullest and freest entrance, and to chase from every crypt, cell and corner the elements of uncleanness and corruption which have a vasted interest in darkness. The architect who constructs a house with damp basement, who makes no provision for pure air and light in the office, school or dwelling, however grand may be its proportions, and handsome its ornamentation and design, is equally guilty of destroying innocent lives as is the man who, through carelessness, propagates small-pox or any other dangerous and loathsome disease: yet this subject is treated with apparent indifference, as if it did not concern us, while it is one of vital interest to every one."

Excepting the preparation for that higher eternal life prepared by Christ for his followers, I can conceive of no subject of such paramount importance to every human being as the preservation of health, which is either good or poor as we obey or violate the laws of sanitary science. Yet we, as dentists, have done, and are doing, very little towards protecting the health of the members of our profession. We take very little thought, if any, upon the subject of dental architecture, for the purpose of improving its defects and lighting, heating and ventilating our offices according to the most improved methods for health and comfort, so as to bring them into harmony with the present knowledge of sanitary science.

We sometimes boast of the progress we have made. We look back with pride and satisfaction (and justly, too,) at the triumphs of modern dental surgery; and in no country has so great a degree of perfection been reached in the dental air as in this. Under the treatment of the skillful dental surgeon we see beauty taking the place of hideous deformity. We see, in many instances, health, restored and life prolonged—results gratifying in the extreme—and yet we fall short of doing the greatest good, to the same extent that we fail in the highest degree attainable to preserve our health and usefulness.

Dentistry is an occupation not conducive to health, and we should, therefore, guard against overtaxing either our physical or nervous system, as well as against assuming constrained and unnatural positions of the body while oporating. Too many operators assume stooping, twisted and constrained positions, compressing the abdominal regions, by leaning upon the chair, causing defective digestion with its usual complications. These should all be avoided. We should stand, or sit if you please, in such positions that every organ has, as far as possible, entire freedom; neither should we approach nearer to the patient than is absolutely necessary to see clearly; we would then not only avoid, to a great extent, breathing the contaminated exaltations, before they are somewhat neutralized, but also save straining the eyes, by fixing them upon an object unnecessary close, which, if persisted in, will sooner or later ruin the best sight. Myopia and asthenopia are both frequently produced in persons whose occupations require very fine sight, who steadfastly look upon a single point till there is a sensation of exhaustion and indistinctness of vision. Myopia is, in a large majority of cases, permanently produced during school days, by holding books too close without resting the eyes—by viewing distant objects. It is almost entirely confined to large cities and towns, where the eyes are seldom fixed upon objects farther than across the street. In the country, where the eyes are not so constantly used for study, and where they are more frequently directed to objects at a distance, this disease is almost unknown.

Asthenopia is a disease more commonly known among dental practitioners, and is also caused by looking at a small object in very close proximity, overworking the muscles, either the internal secti or muscles of accommodation, by using them in a poor light, or otherwise exhausting and injuring them.

The best way to avoid these diseases is to have a strong, steady light from the north, and to work only while you can see clearly and distinctly, and frequently rest the eye by looking around the room, or at some distant object out of the window or across the street. Never look steadfastly upon a single point until there is a sensation of exhaustion and indistinctness of vision. Happily however, since we make use of the rubber dam there is seldom, if ever, any great need to overwork the eyes by a too prolonged straining, for with this secure around our tooth, we can take time to rest the eyes as necessity demands.

The highest aim of the dental profession is the preservation of the natural teeth; and we, as members of the profession, fall short of accomplishing the greatest good in proportion as we in the highest degree attainable fail to preserve our health and usefulness. However perfect we may be in the theory, or even practice of medicine; however skilful we may be in surgery or operative dentistry, or ingenious in supplying artificial teeth or other mechanical appliances, we are incapacitated from performing the very best professional services for our patients, just in proportion as we have physically or mentally depreciated by neglecting the laws of life and health.—*Transactions of Pennsylvania State Dental Society.*

